# Visual outcome of 25-gauge microincision vitrectomy surgery in diabetic vitreous haemorrhage

**DOI:** 10.12669/pjms.315.7182

**Published:** 2015

**Authors:** Burhan Abdul Majid Khan, Syed Fawad Rizvi, Syed Asaad Mahmood, Washoo Mal, Shakir Zafar

**Affiliations:** 1 Dr. Burhan Abdul Majid Khan, FCPS (Ophth). Ophthalmologist, LRBT Free Base Eye Hospital, Korangi 2 ½, Karachi, Pakistan; 2 Dr. Syed Fawad Rizvi, FCPS (Ophth), MCPS (Ophth). Chief Consultant Ophthalmologist, LRBT Free Base Eye Hospital, Korangi 2 ½, Karachi, Pakistan; 3 Dr. Syed Asaad Mahmood, MBBS, FCPS-I (Ophth). Resident, LRBT Free Base Eye Hospital, Korangi 2 ½, Karachi, Pakistan; 4 Dr. Washoo Mal, FCPS (Ophth). Assistant Ophthalmologist, LRBT Free Base Eye Hospital, Korangi 2 ½, Karachi, Pakistan; 5 Dr. Shakir Zafar, FCPS (Ophth), MCPS (Ophth). Consultant Ophthalmologist, LRBT Free Base Eye Hospital, Korangi 2 ½, Karachi, Pakistan

**Keywords:** 25-gauge micro incision vitrectomy surgery, Vitreous haemorrhage, Diabetic retinopathy

## Abstract

**Objective:**

To assess the visual outcome and complications of 25-gauge micro incision vitrectomy surgery (MIVS) in diabetic vitreous haemorrhage.

**Methods:**

This Quasi Experimental study was conducted at LRBT, Tertiary eye care hospital Karachi, from February 2012 to January 2013. Sixty eyes of sixty patients with uncontrolled type II diabetes mellitus (DM) were included. There were 43 (71.7%) males and 17 (28.3%) females. Age range was 40 – 60 years. All randomly selected patients underwent 25-gauge sutureless micro incision vitrectomy surgery for diabetic vitreous haemorrhage. Main outcomes measured were best corrected visual acuity (BCVA) assessed with logMAR and post-operative complications. Follow ups were at one day, one week, one month, three months and six months post-operatively.

**Result:**

Best corrected visual acuity (BCVA) gradually improved in majority of subjects in each subsequent follow up visit. Preoperative visual acuity was 1.023 ±0.226 logMAR, which was improved after final follow up to 0.457±0.256 and P-value was < 0.001. Five patients developed recurrent vitreous haemorrhage during study period, one patient developed cataract (1.7%), one (1.7%) had ocular hypotony defined as intraocular pressure < 5 mmHg and one (1.7%) developed endophthalmitis.

**Conclusion:**

25-gauge micro incision vitrectomy surgery (MIVS) is an effective sutureless parsplana vitrectomy surgery which has good visual outcome in diabetic vitreous haemorrhage with minimum manageable complications.

## INTRODUCTION

It has been found that prevalence of proliferative diabetic retinopathy (PDR) in Pakistan is 2.65–5%.^1^,^2^ One of the major causes of visual impairment in patients of proliferative diabetic retinopathy is vitreous haemorrhage but due to emergence of vitrectomy system and rapid advancement in surgical techniques for posterior segment pathologies there is dramatic recovery of visual acuity after surgery. David Kasner in 1969 introduced the initial description of major vitreous surgery, detailing the excision and removal of the vitreous using sponge and scissors under an open sky technique. Machemer et al introduced parsplana vitrectomy in 1971.^3^

For PDR, pan retinal photocoagulation (PRP) is the primary treatment, but for more complicated cases, such as those with associated vitreous haemorrhageand/or tractional retinal detachment, a vitrectomy surgery is performed.^4^,^5^ Closed surgical system for removal of vitreous with controlled intraocular pressure was provided by the technique described by Machemer. The device used was a 17-gauge (1.42mm) instrument combined with vitreous cutter, infusion and aspiration. In 1975, O’Malley and Heinz developed the first 20-gauge 3-port parsplana vitrectomy but by then, a number of complications had become apparent. One of the major problem was the development of iatrogenic retinal breaks specifically at sclerotomy site. Improvement in technique and instruments design quickly led to the development of 3-port vitrectomy system with light weight, reusable, pneumatic and electric cutters.

Dutch Ophthalmic Research centre (DORC) working with Klaus Eckardt presented the first 23-gauge vitrectomy system in 2005. GlidoFujii introduced a sutureless transconjunctival 25-gauge micro incision vitrectomy system (MIVS) in 2002.^6^ Micro incision vitrectomy surgery without peritomy and suturing facilitates early visual recovery while decreasing patient discomfort, operating time, post-operative inflammation, ocular surface irregularities and surgically induced astigmatism.^7^-^9^ The second generation trocar cannulas require a lower insertion force, so stabilization of the eye is no longer a problem.^10^

The rationale of this study is to look postoperative visual outcome and complications after 25-gauge micro incision vitrectomy surgery in patients with diabetic vitreous haemorrhage.

## METHODS

After obtaining approval from the hospital ethics committee, this prospective, non-comparative, interventional study was carried out at LRBT tertiary eye hospital Karachi from February 2012 to till January 2013. Sixty patients were admitted from outpatient clinics and only one eye per patient was enrolled. Patients were selected using non probability convenience sampling technique. Included in the study were adults of both genders who were known cases of diabetic retinopathy and had non resolving vitreous haemorrhage. Those excluded were patients with any type of retinal detachment, glaucoma, uveitis, cataract and history of previous ocular surgery.

Before surgery, the procedure along with its risks and benefits was explained to the patient and a written informed consent was taken. Data collected included patients’ age, gender, date of admission, date of operation, date of discharge, investigations, and any post-operative complication, specifically hypotony, corneal edema, cataract, eye discomfort, conjunctival scarring and endophthalmitis. In all patients preoperative ocular examination consisted of assessment of visual acuity (logMAR chart), ocular adnexa, anterior segment examination, crystalline lens for opacities (cataract), intraocular pressure measured using Goldmann applanation tonometer (Haag Streit AT 900), dilated fundal examination with +90 D lens on a slit lamp biomicroscope and via indirect ophthalmoscopy. Systemic assessment included measurement of blood pressure, fasting blood sugar and HbA1c.

Equipment used was Zeiss microscope with EIBOS 2 attachment for non-contact fundus viewing (Haag-Streit Surgical GmbH), Constellation vitrectomy machine (Alcon Laboratories Inc.), and a 25-gauge system (infusion cannula, fibre optic endoilluminator, vitrectomy cutter, trocar cannulas set, endolaser probe). All patients were operated by a single surgeon with over 10 years of experience in posterior segment surgery. A 25-gauge 3-port vitrectomy setup was used for all cases. Retrobulbar injection (2ml of lidocaine 2% and 2ml of bupivacaine 0.75%) was given for local anaesthesia in all cases. After strict aseptic patient preparation and draping, scleral tunnels were formed by self-retaining trocar cannulae inserted 30 degree obliquely transconjunctivally at inferotemporal, superonasal and superotemporally about 4.00mm away from the limbus. The infusion line was connected to inferotemporal cannula. The infusion line was opened that prevented hypotony while making second and third sclerotomy ports. The two superior cannule were used for illumination and vitrectomy cutter. After complete vitrectomy, pan retinal photocoagulation was done using the endolaser probe. In the end, superior cannulae were removed while observing the repositioning of conjunctiva covering the sclerotomies. Finally inferotemporal cannula with infusion line was removed and repositioning of conjunctiva was confirmed.

Postoperative follow ups were at one day, one week, one month, three months and finally at six months. Visual acuity was measured using a logMAR chart by an experienced optometrist and was followed by refraction (where needed) to assess the BCVA. Postoperative complications were screening for during all follow up visits and those that were encountered were noted down in the records and managed accordingly. Best corrected visual acuity data was analyzed using IBM SPSS Statistics 21 by applying paired sample t- test with 95% confidence interval. A p-value of ≤ 0.05 was considered statistically significant.

## RESULTS

Data of sixty eyes of sixty patients after 25-gauge micro incision vitrectomy surgery was analyzed. Age range was 40-60 years (52.43±6.7), with 43 (71.7%) males and 17 (28.3%) females. The pre-operative and post-operative BCVA are shown in Table-I. The pre-operative BCVA was compared with post-operative BCVA at day one, month one and month 6 follow-up. The paired sample t-test with 95% confidence interval showed a p-value < 0.001 for all 3 pairs.

**Table-I:** Pre- and post-operative best corrected visual acuity (BCVA).

BCVA (logMAR)	Pre-op	Post-op day 1	Post-op week 1	Post-op month 1	Post-op month 3	Post-op month 6
Mean	1.023	0.768	0.645	0.515	0.473	0.457
±Std. Deviation	±0.226	±0.132	±0.148	±0.183	±0.253	±0.257

Post-operative complications are summarized in Table-II. These were managed on a case-to-case basis. Those who had recurrent vitreous haemorrhage were re-operated after a few months to clear the vitreous haemorrhage through 25 gauge MIVS.

**Table-II:** Complications during 6 months follow-up.

Complication	Frequency	Percent
Cataract	1	1.7
Endophthalmitis	1	1.7
Hypotony	1	1.7
Vitreous Haemorrhage	5	8.3
Total	60	100

## DISCUSSION

Advantages of 25-gauge micro incision vitrectomy includes avoidance of conjunctival peritomy, less post-operative discomfort reported by the patients, less use of irrigation solution because smaller 25-gauge instruments has lower aspiration volumes and this theoretically reduce postoperative inflammation, and less post-operative astigmatism.^11^,^12^ The results of this study show significant improvement in BCVA at six months after vitrectomy. In addition, the greatest degree of improvement in BCVA was already present at the time of hospital discharge (post-operative day 1) and by the end of the first post-operative month, the patients had already recovered the majority of their potential BCVA with small improvements continuing at least up to six months post-operatively. A similar study by Sato et al. shows a BCVA of 0.68±0.57 at the time of hospital discharge and BCVA of 0.54±0.63 at six months after vitrectomy, which is comparable to this study.^13^

With the use of 25-gauge micro incision vitrectomy surgery, there is no need of conjunctival peritomy and this shortens the operating time as well as helps in the early visual recovery due to lesser manipulation compared with larger gauge vitrectomy. However, due to the sutureless nature of 25-gaugeMIVS, the risk of post-operative hypotony and consequently the risk of post-operative endophthalmitis is increased.^14^,^15^ To minimize this risk, an oblique incision is used to create the transconjunctival sclerotomies, and if a leaky wound is found, transconjunctival sutures are used to close the sclerotomy.^16^ Literature shows increase incidence of postoperative hypotony in sutureless vitrectomy.^6^,^17^ Hypotony (defined as ≤ 5mmHg intraocular pressure) occurred in one patient (1.7%) in this study and which was normalized in two weeks with use of topical steroids and cycloplegics. Woo SJ et al. noted postoperative hypotony in 11.3% after MIVS.^18^ Kunimote et al. in one large retrospective series has shown the incidence of endophthalmitis after sutureless 25-gauge MIVS to be twelve times higher than conventional 20-gauge vitrectomy.^19^ However, in subsequent case series of Hu Ay et al. and Parolini B et al., no increased incidence of endophthalmitis was seen in 25-gauge and 23-gauge MIVS compared with 20-gauge vitrectomy.^20^,^21^ In the study by Sato et al. no cases of endophthalmitis were reported.^13^

Recurrent vitreous haemorrhage occurred in five cases (8.3%) and this is probably due to underlying basic pathology of proliferative diabetic retinopathy. In this study, a single case developed endophthalmitis which was successfully treated with intra-vitreal, topical and systemic antibiotics.

## CONCLUSION

25-gauge MIVS is an effective sutureless pars plana vitrectomy surgery which has good visual outcome in diabetic vitreous haemorrhage with minimum and manageable complications. 
